# Supplementary material for: Colon Bioaccessibility under In Vitro Gastrointestinal Digestion of Different Coffee Brews Chemically Profiled through UHPLC-Q-Orbitrap HRMS
Source: Foods. 2021 Jan 17;10(1):179. doi: 10.3390/foods10010179 (PMC7829986; doi:10.3390/foods10010179)
Supplement: Supplementary file 1 [file foods-10-00179-s001.pdf]

Supplementary materials:

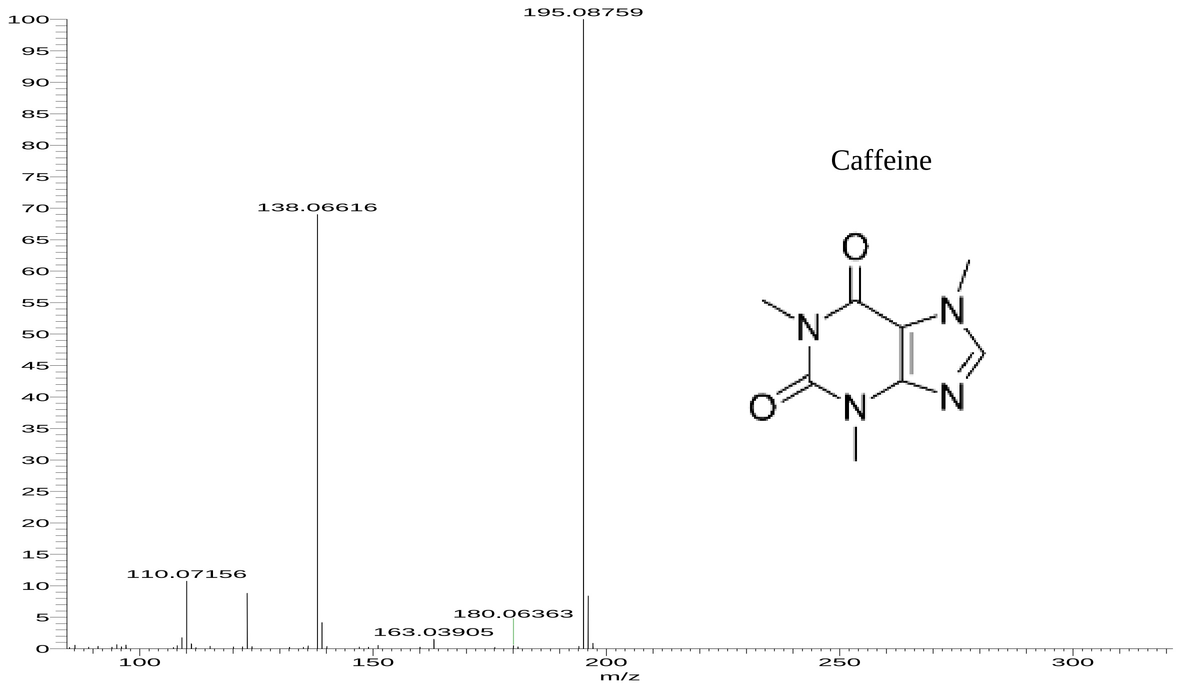

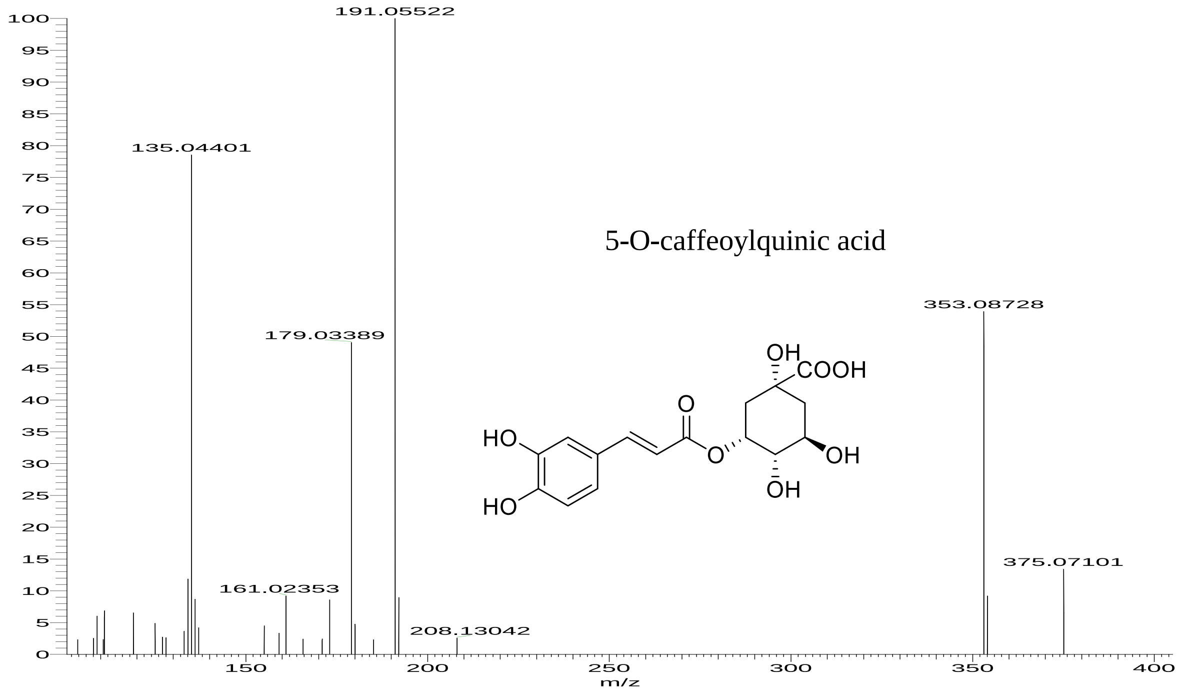

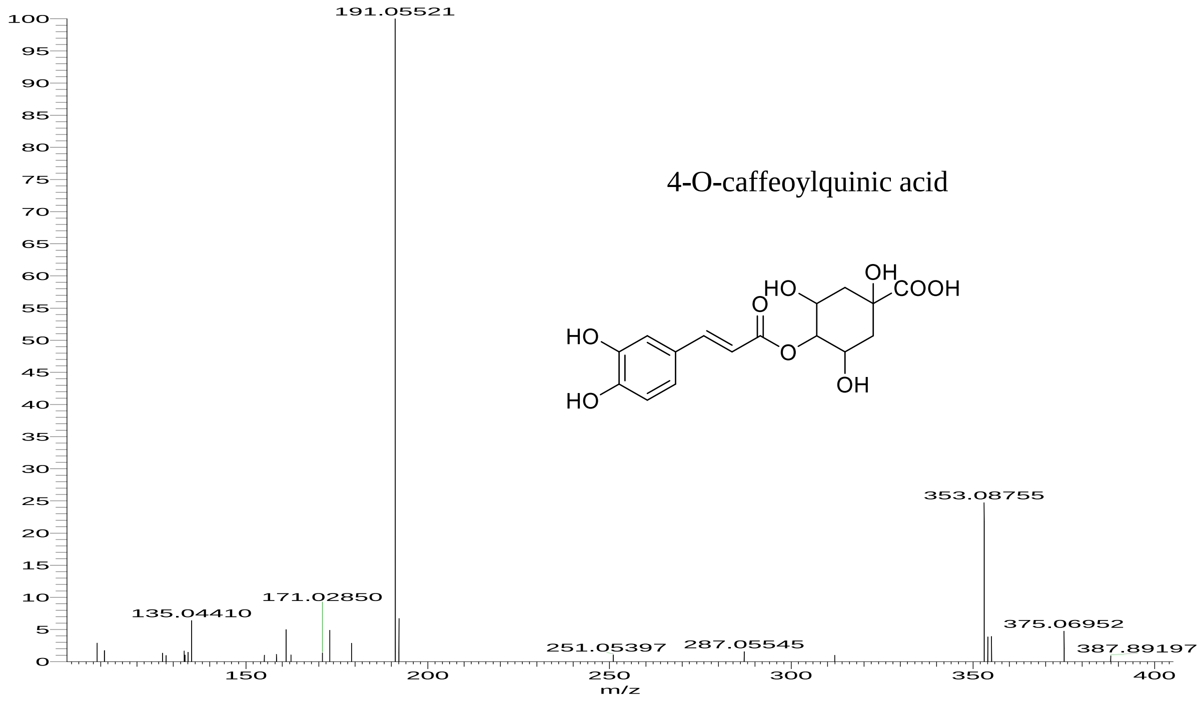
4-O-caffeoylquinic acid
191.05521
353.08755
135.04410
171.02850
375.06952
251.05397
287.05545
387.89197
m/z
HO
OH
COOH
O
HO
O
OH
HO

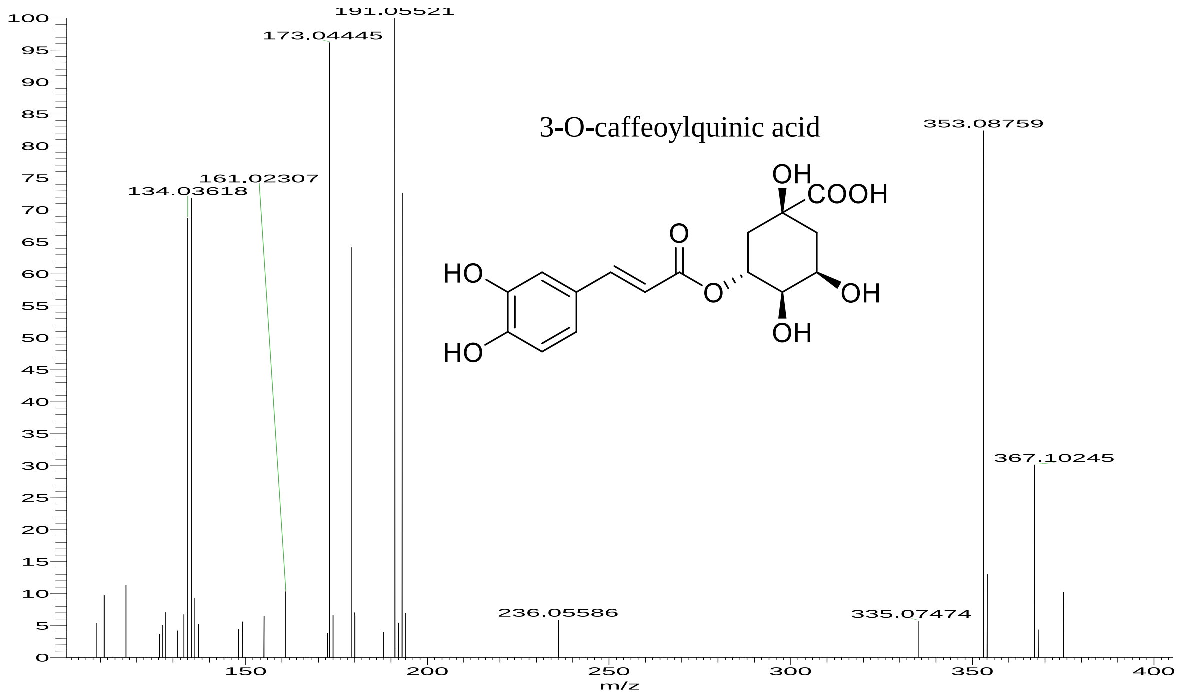
3-O-caffeoylquinic acid
191.05521
173.04445
353.08759
161.02307
134.03618
367.10245
236.05586
335.07474
m/z
OH
COOH
O
HO
O
OH
OH
HO

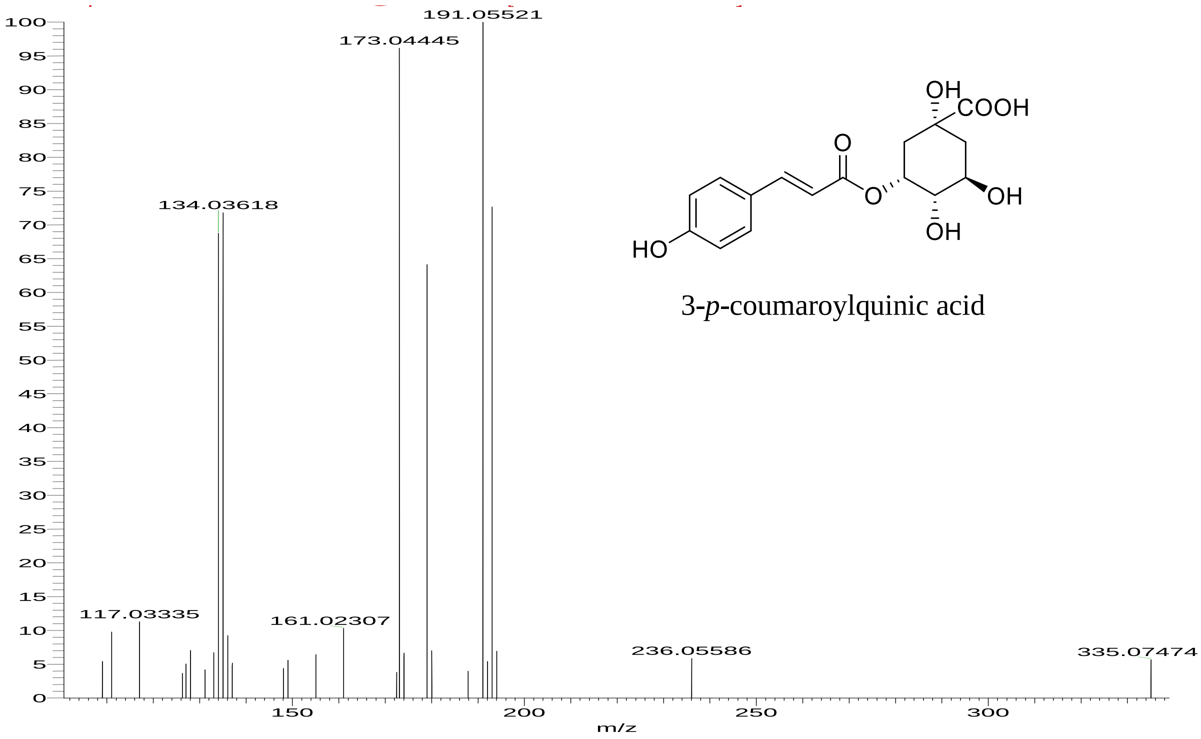
191.05521
173.04445
134.03618
117.03335
161.02307
236.05586
335.07474
m/z
3-p-coumaroylquinic acid

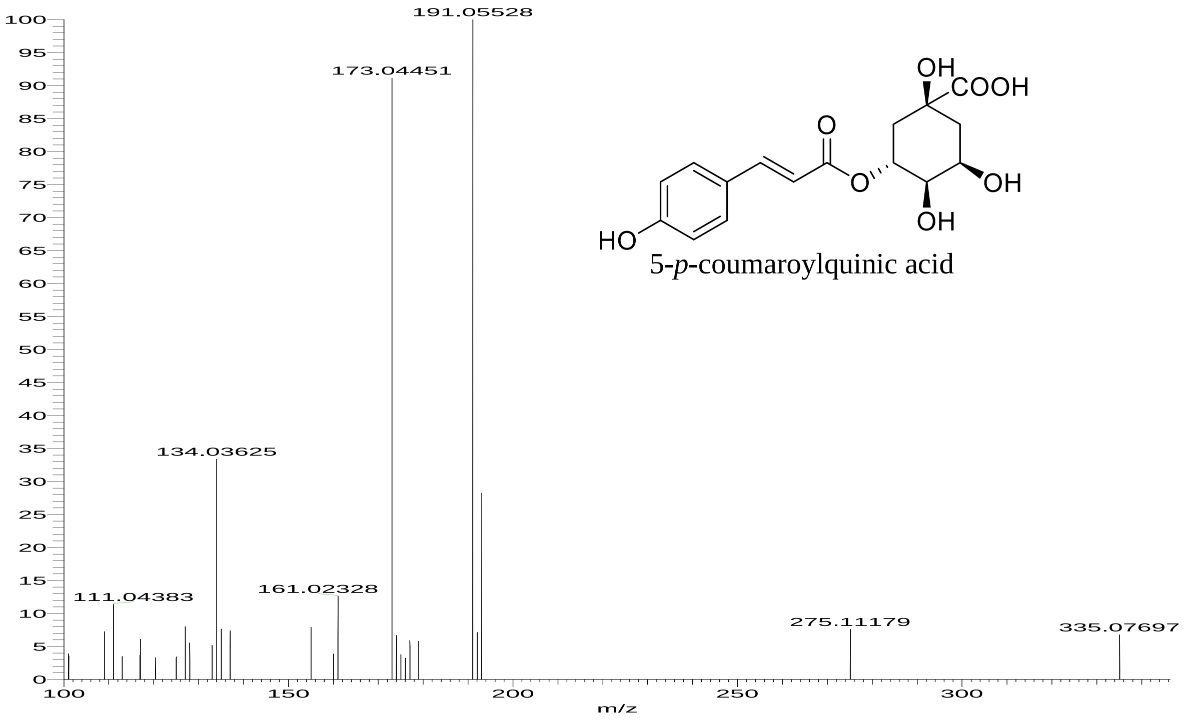
191.05528
173.04451
134.03625
111.04383
161.02328
275.11179
335.07697
m/z
5-p-coumaroylquinic acid

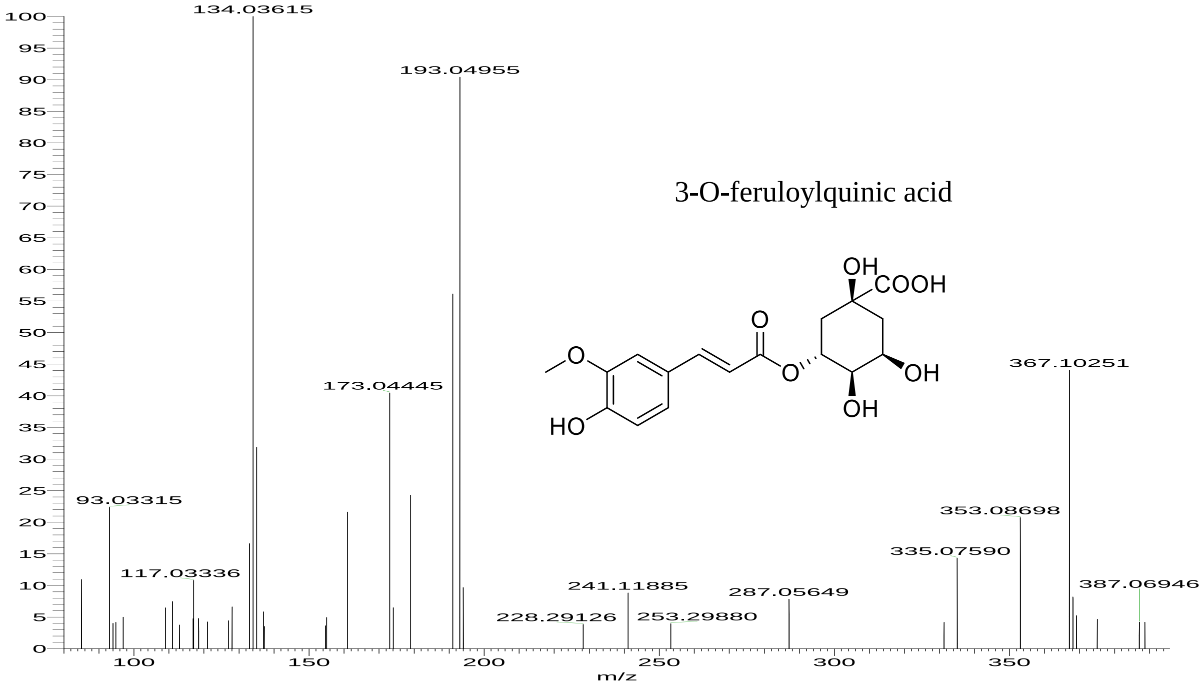

134.03615
193.04955
3-O-feruloylquinic acid
OH
COOH
O
O
OH
OH
HO
173.04445
367.10251
93.03315
353.08698
335.07590
117.03336
241.11885
287.05649
387.06946
228.29126
253.29880
m/z

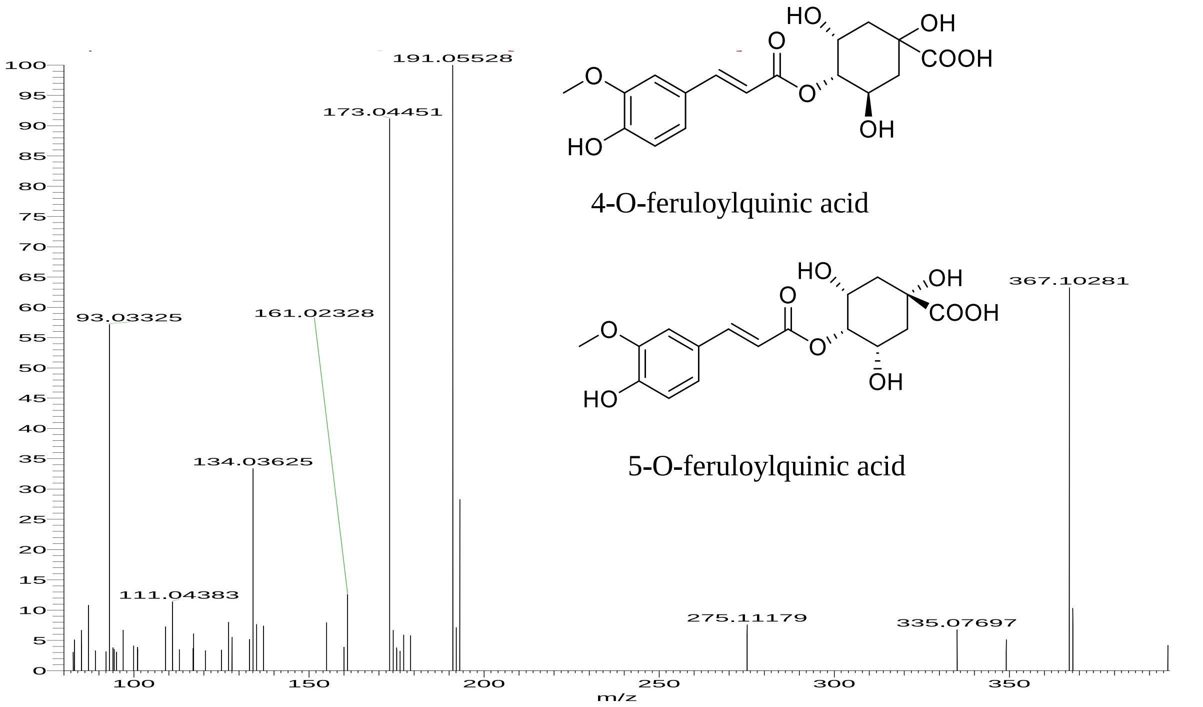

191.05528
173.04451
HO
OH
O
COOH
O
O
OH
HO
4-O-feruloylquinic acid
HO
OH
COOH
O
O
OH
HO
93.03325
161.02328
367.10281
5-O-feruloylquinic acid
134.03625
111.04383
275.11179
335.07697
m/z

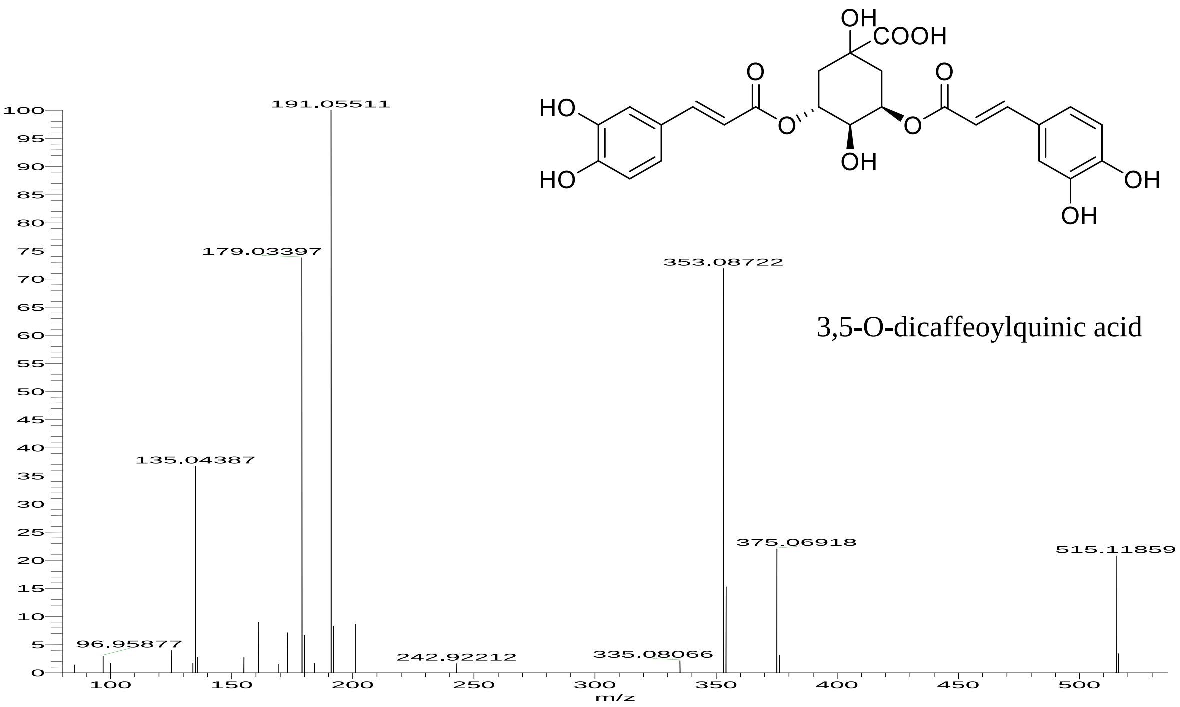

3,5-O-dicaffeoylquinic acid

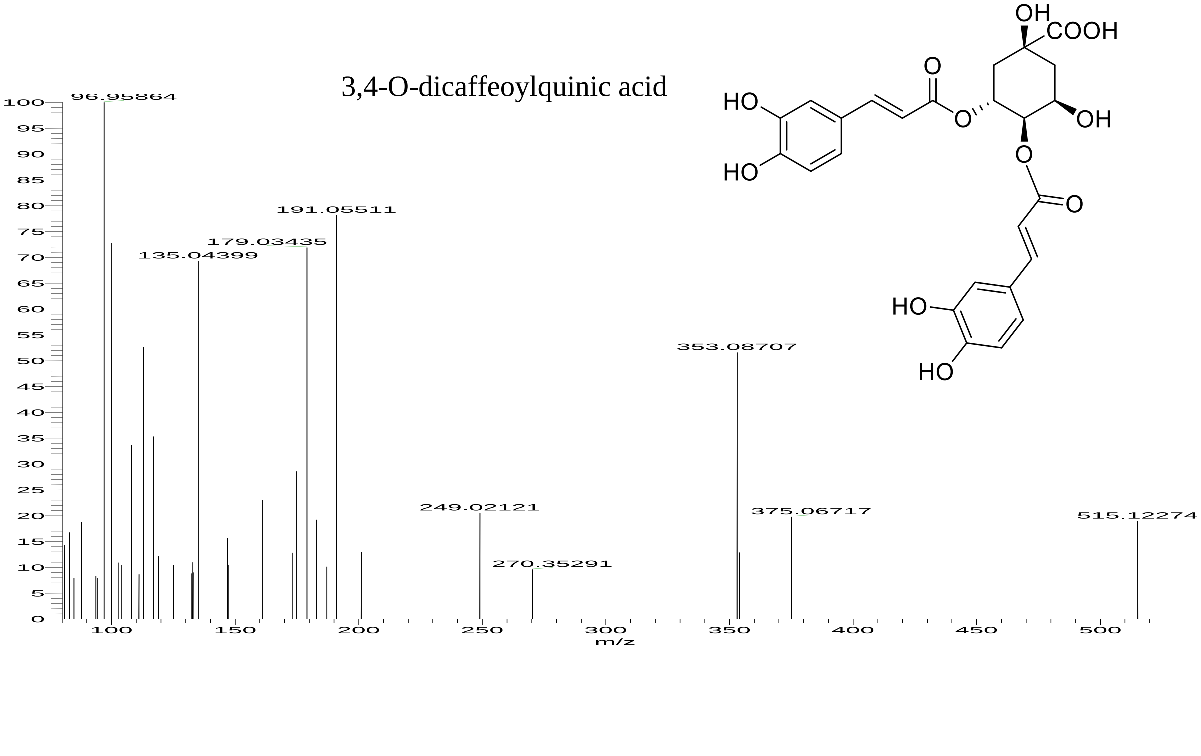

3,4-O-dicaffeoylquinic acid

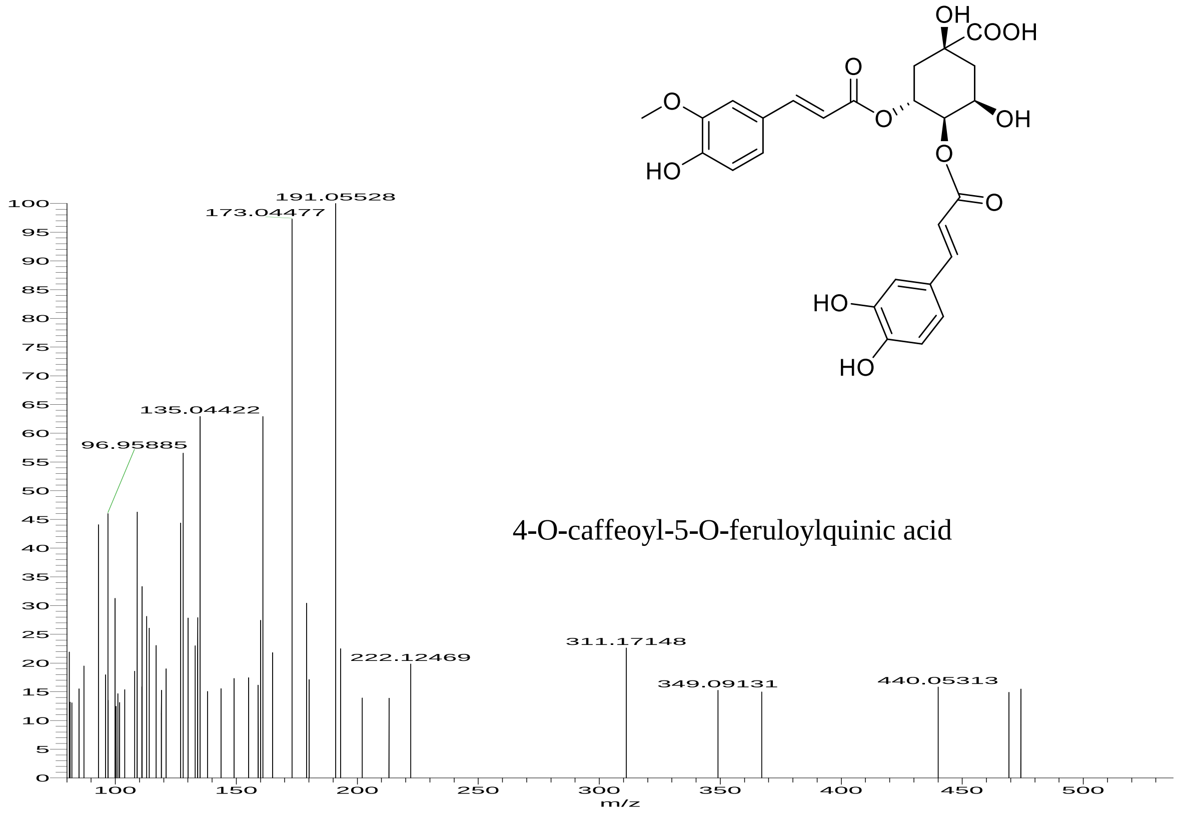

4-O-caffeoyl-5-O-feruloylquinic acid

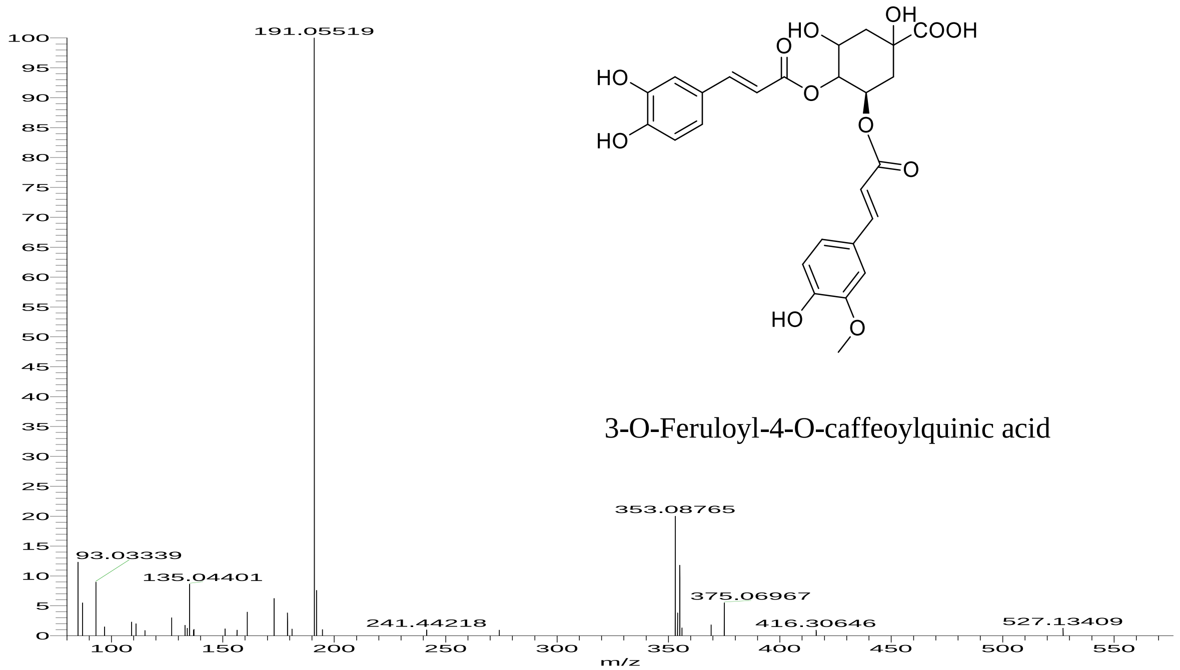

3-O-Feruloyl-4-O-caffeoylquinic acid

Supplementary Figure S1. MS/MS mass spectra of investigated compounds (n = 12) in assayed coffee brews.

Table S1. Composition of stock solutions of simulated digestion fluid.

Salt solution	Stock concentration (mol/L)	SSF (pH 7)		SGF (pH 3)		SIF (pH 7)	
		mL of Stock added to prepare 0.4 L (mL)	Final salt concentration in sample (mmol/L)	mL of Stock added to prepare 0.4 L (mL)	Final salt conc. in sample (mmol/L)	mL of Stock added to prepare 0.4 L (mL)	Final salt conc. in sample (mmol/L)
KCl	0.5	15.1	15.1	6.9	6.9	6.8	6.8
KH_2PO_4	0.5	3.7	1.35	0.9	0.9	0.8	0.8
$NaHCO_3$	1	6.8	13.68	12.5	25	42.5	85
NaCl	2	-	-	11.8	47.2	9.6	38.4
$MgCl_2(H_2O)_6$	0.15	0.5	0.15	0.4	0.12	1.1	0.33
$NH_4(CO_3)_2$	0.5	0.06	0.06	0.5	0.5	-	-
$CaCl_2(H_2O)_2$	0.3	-	1.5	-	0.15	-	0.6

Table S2. HMWM content in the analyzed coffe brews.

Samples	Melanoidins (mg/mL) ± SD
Espresso	6.23 ± 0.41
Americano	0.71 ± 0.09
Instant coffee	5.78 ± 0.52

(A)

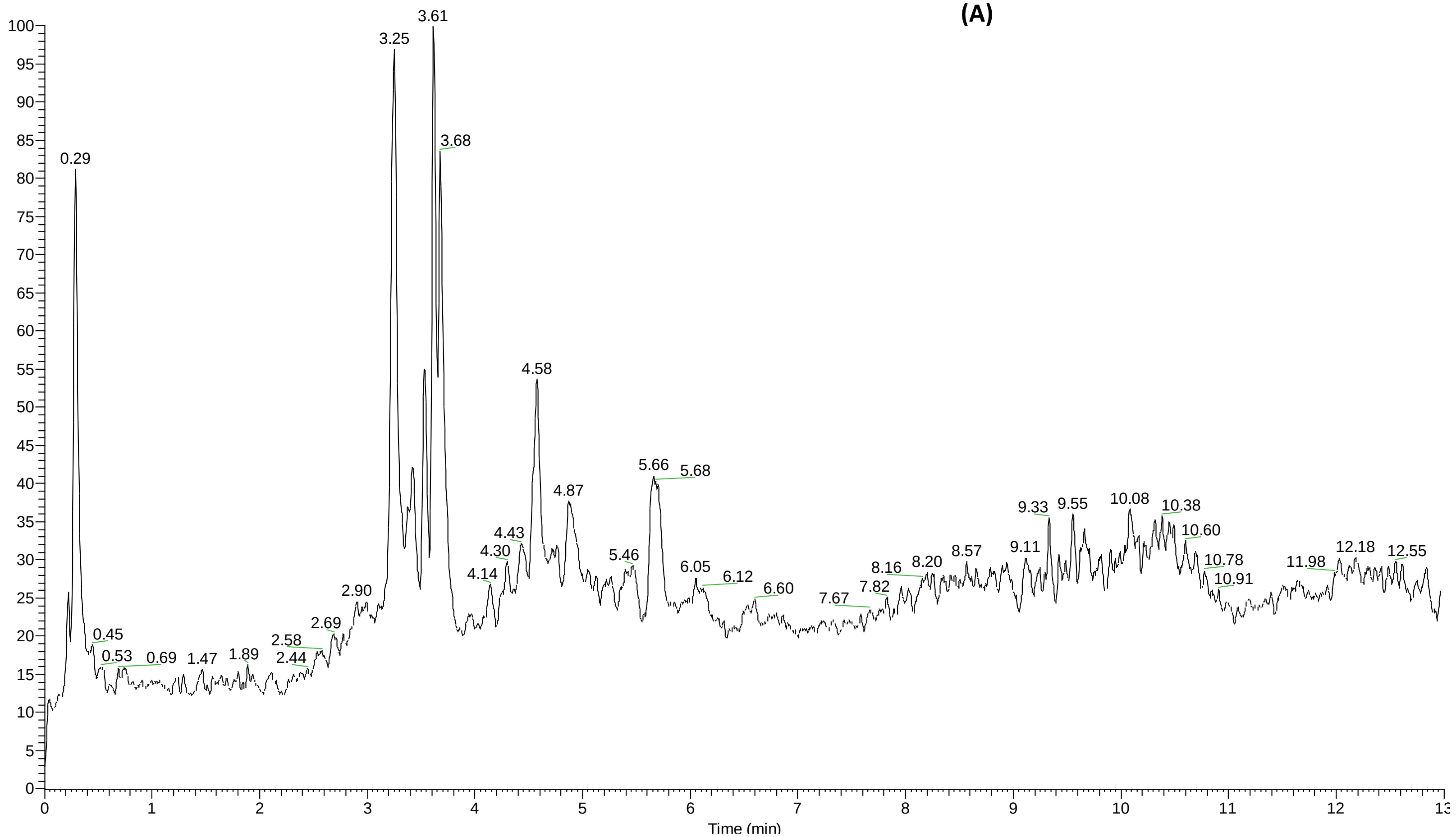

0.29
0.45
0.53
0.69
1.47
1.89
2.58
2.44
2.69
2.90
3.25
3.61
3.68
4.14
4.30
4.43
4.58
4.87
5.46
5.66
5.68
6.05
6.12
6.60
7.67
7.82
8.16
8.20
8.57
9.11
9.33
9.55
10.08
10.38
10.60
10.78
10.91
11.98
12.18
12.55
Time (min)

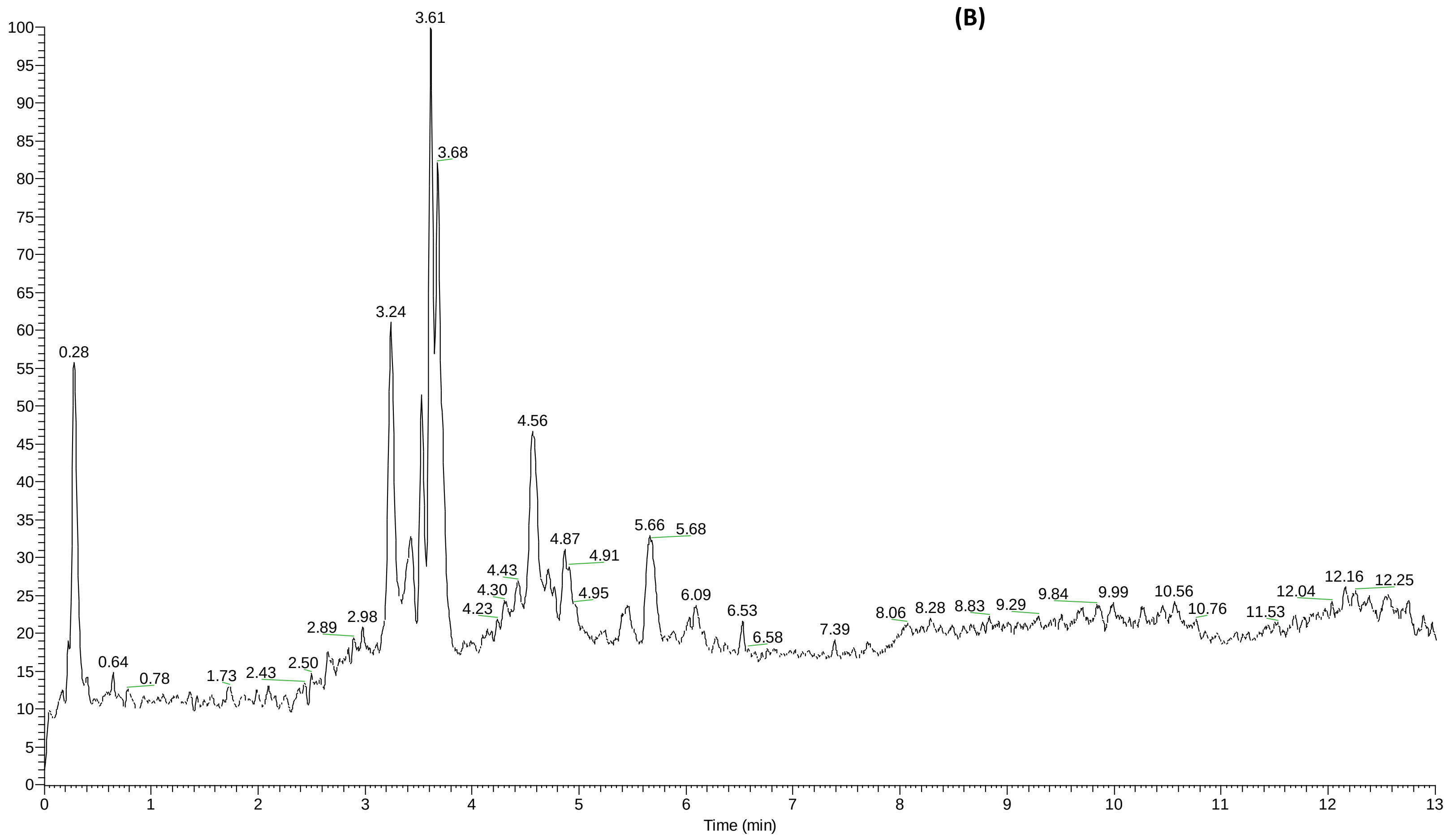
(B)
Time (min)
0.28
0.64
0.78
1.73
2.43
2.50
2.89
2.98
3.24
3.61
3.68
4.23
4.30
4.43
4.56
4.87
4.91
4.95
5.66
5.68
6.09
6.53
6.58
7.39
8.06
8.28
8.83
9.29
9.84
9.99
10.56
10.76
11.53
12.04
12.16
12.25

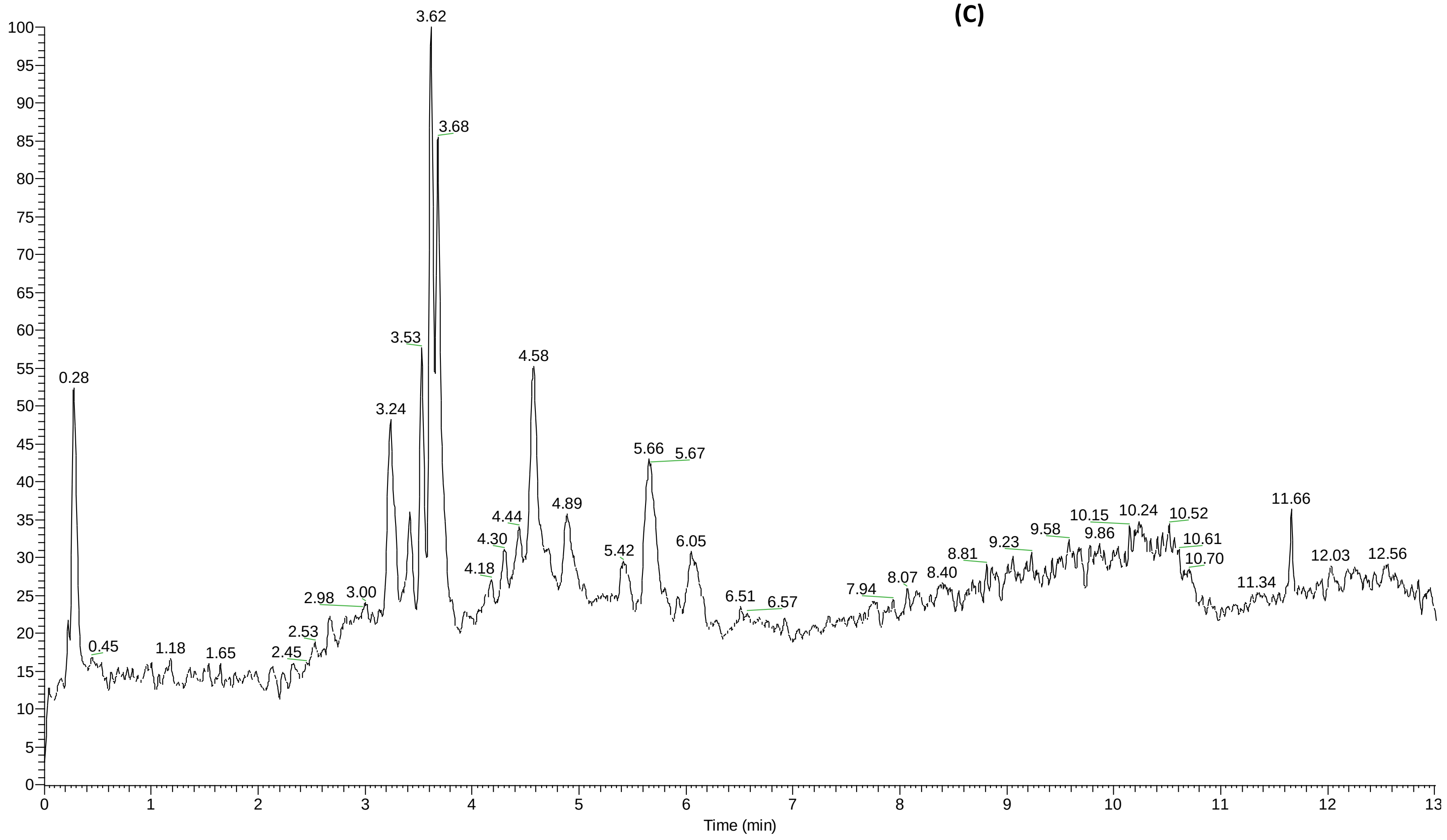

Supplementary Figure S2. Total Ion Chromatogram (TIC) of espresso (A), americano (B), and instant coffee brews (C) through UHPLC-Q-Orbitrap HRMS.
